# Mechanosensitive changes in the expression of genes in colorectal cancer-associated fibroblasts

**DOI:** 10.1038/s41597-023-02233-9

**Published:** 2023-06-02

**Authors:** Bashar Emon, You Jin Song, M. Saddam H. Joy, Mounisha V. Kovour, Kannanganattu V. Prasanth, M. Taher A. Saif

**Affiliations:** 1 Dept. of Mechanical Science & Engineering, University of Illinois at Urbana-Champaign, Urbana, IL USA; 2 Dept. of Cell and Developmental Biology, University of Illinois at Urbana-Champaign, Urbana, IL USA; 3 Cancer Center at Illinois, University of Illinois at Urbana-Champaign, Urbana, IL USA

**Keywords:** Cancer microenvironment, Cancer genomics, Gene ontology, Gene expression, Computational biophysics

## Abstract

Most solid tumors become stiff with progression of cancer. Cancer Associated Fibroblasts (CAFs), most abundant stromal cells in the tumor microenvironment (TME), are known to mediate such stiffening. While the biochemical crosstalk between CAFs and cancer cells have been widely investigated, it is not clear if and how CAFs in stiffer TME promote metastatic progression. To gather insights into the process, we controlled the mechanical stiffness of the substrates and collected gene expression data with human colorectal CAFs. We cultured human primary CAFs on 2D polyacrylamide hydrogels with increasing elastic modulus (E) of 1, 10 and 40 kPa, and performed genome-wide transcriptome analyses in these cells to identify expression levels of ~16000 genes. The high-quality RNAseq results can be an excellent data-source for bioinformatic analysis for identifying novel pathways and biomarkers in cancer development and metastatic progression. With thorough analysis and accurate interpretation, this data may help researchers understand the role of mechanical stiffness of the TME in CAF-cancer cell crosstalk.

## Abstract (summary)

**Table:** 

Design Type(s)	solid tumor development model • biophysical progression in colorectal cancer • transcription profiling by high throughput sequencing design
Measurement Type(s)	transcription profiling assay
Technology Type(s)	RNA sequencing
Factor Type(s)	substrate mechanical stiffness • cancer progression stage
Sample Characteristic(s)	Human (*Homo sapiens*) • colorectal cancer (CRC)• cancer associated fibroblast

## Background & Summary

Cancer metastasis is a complex process that involves dynamic crosstalk between cancer and stromal cells. Only recently have we begun to understand that tumor microenvironment (TME) is a key mediator of such interactions. However, the precise mechanism by which TME influences metastasis is still not well understood. Both physical and chemical properties of TME play major roles in prompting the cancer cells to undergo epithelial to mesenchymal transition (EMT) that leads to metastasis^1,2^. Among many factors of the TME, the most prominent is increasing stiffness of solid tumors^3–5^. Extracellular matrix (ECM) stiffness and/or tumor rigidity are known to facilitate pro-metastatic progression in many types of cancer such as breast and colorectal cancer (CRC)^2,6^. Here we share transcriptome resources that researchers can utilize for understanding the role of TME stiffness in crosstalk between stromal fibroblasts and cancer cells.

**Fig. 1:**
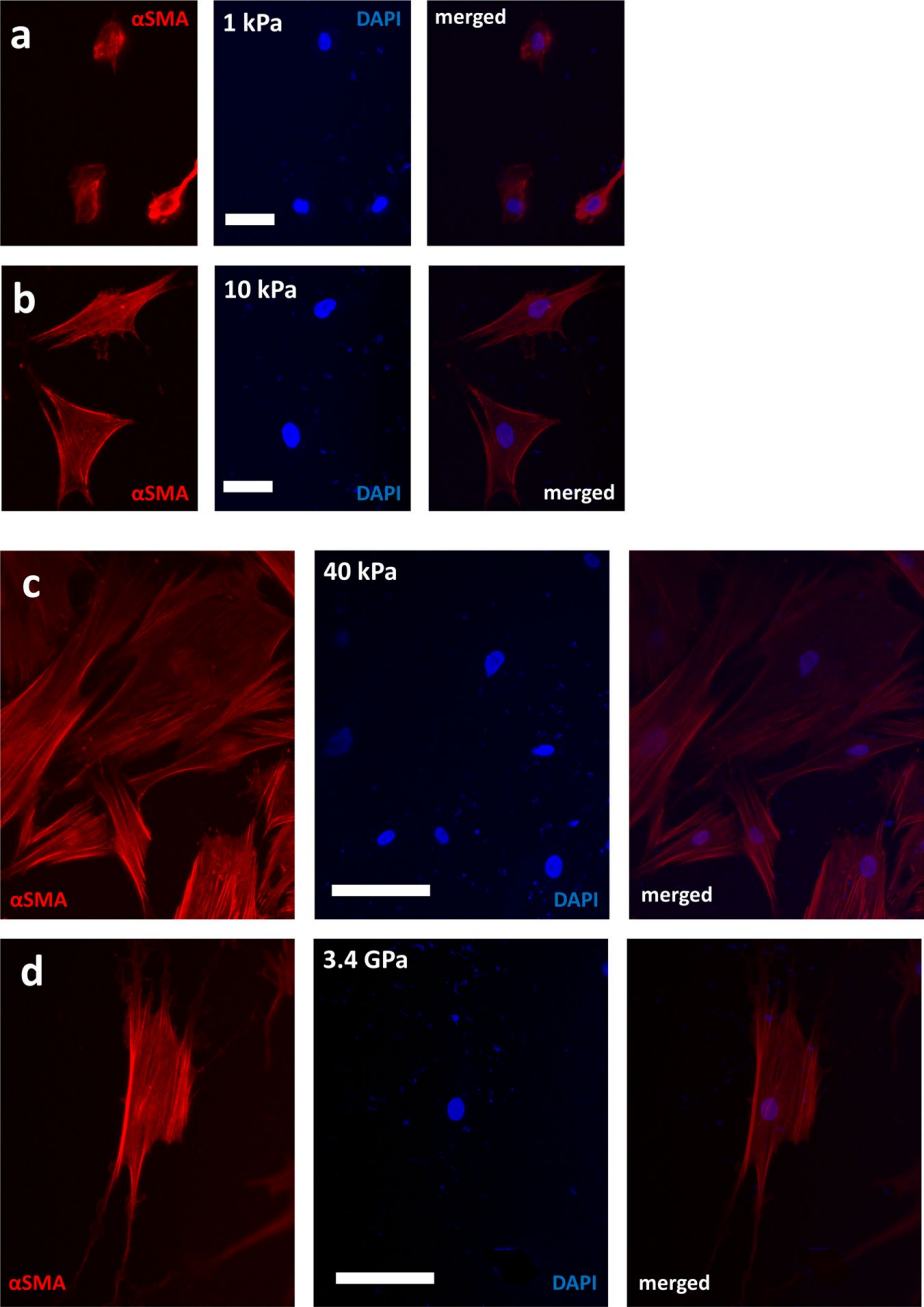
Morphologies/phenotypes of CAFs on substrates of (**a**) 1 kPa (**b**) 10 kPa, (**c**) 40 kPa and (**d**) 3.4 GPa (plastic) elastic modulus. Immunocytochemistry of α-Smooth Muscle Actin (red) and nuclei (blue) shows cell spread, stress fibers and nuclear shapes on different stiffness. Images are maximum intensity projections from a z-stack of confocal images. Scale bars: 50 um.

Among the different types of stromal cells, CAFs are considered to be most important; because they are a key player in tumorigenesis and constitute the majority of the stromal population^7,8^. While genomic heterogeneity of cancer cells has been extensively studied, we know little about the heterogeneity of stromal cells. It is also not clear how the stromal population diversifies as the TME evolves with time. To address this gap, we focused on CAFs (CAF05, human colorectal primary CAFs from Neuromics) in this study to explore how increased substrate stiffness modulates their gene expressions that potentially facilitate metastatic progression. PolyA + paired-end RNA-seq was performed in biological duplicates from CAFs grown on polyacrylamide hydrogel (PA gel) substrates of 1 kPa, 10 kPa and 40 kPa elastic modulus. To represent typical *in vitro* culture condition, CAFs were also collected from polystyrene substrates (elastic modulus of 3.4 GPa). Figure 1 shows CAFs cultured on substrates with different mechanical stiffness. Currently, we do not have publicly available datasets for colorectal CAF transcriptome characterized with increasing substrate stiffness.

**Fig. 2:**
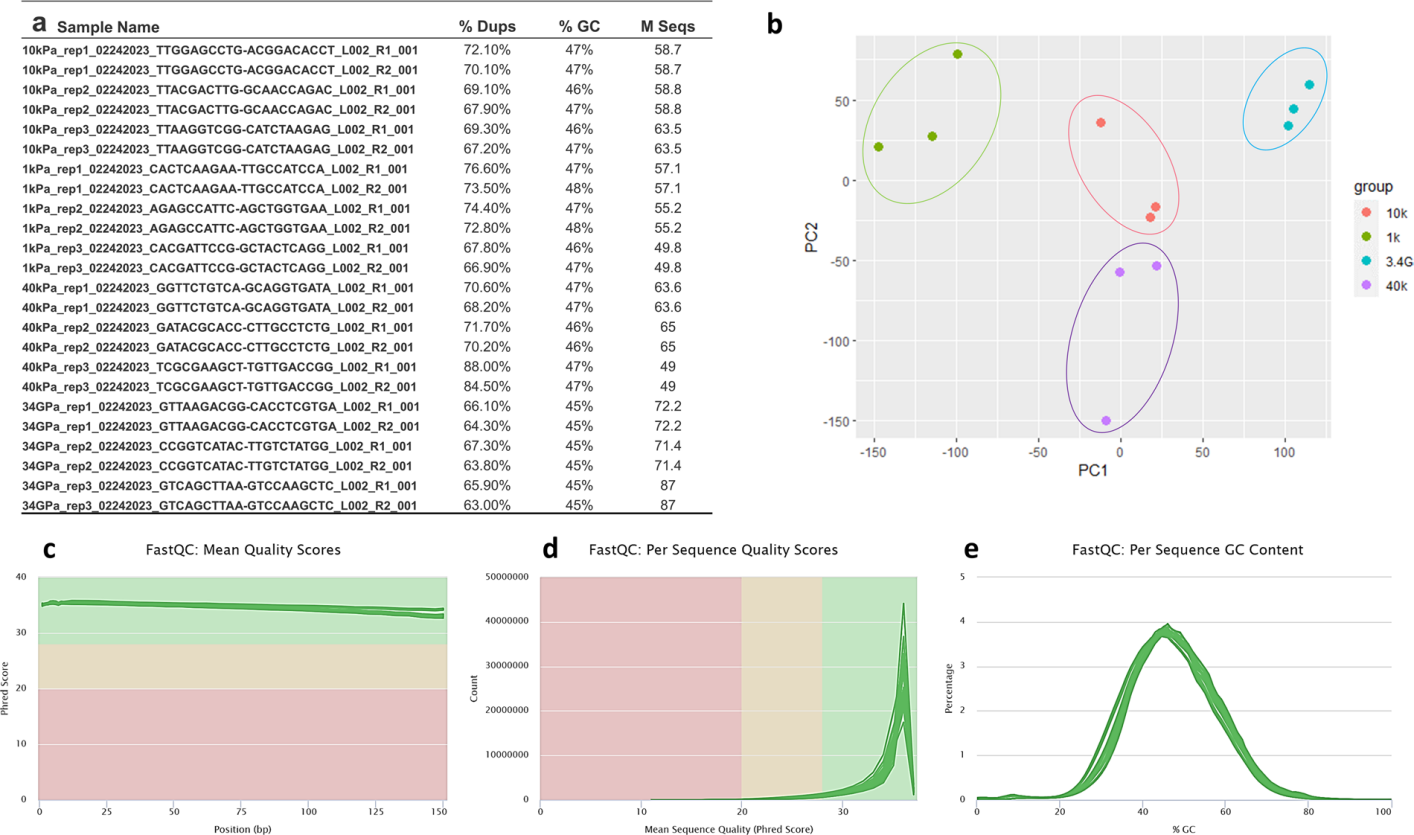
Quality assessment by FastQC shows high quality sequences for all samples. (**a**) General quality statistics of each RNA-seq sample from FastQC. (**b**) Principal Component Analysis (PCA) showing the variations between the RNA-seq samples. (**c**) Sequence quality score per base from FastQC. y-axis shows the mean Phred score, wherein Phred score >28 was defined as “very good quality”. x-axis shows the base position in the read. (**d**) Quality score per sequence from FastQC. y-axis shows the number of reads and x-axis shows the Phred score. (**e**) GC content per sequence.

We have utilized high-precision sequencing technology (RNA-seq) to characterize gene expressions. Quality control and validation of the data is presented in Fig. 2. All relevant metrics indicate that the RNA-seq and analysis data is of high quality. This genome-wide transcriptome dataset may be mined by the research community for further studies. With detailed analysis and accurate interpretation, the data may help find novel pathways that get influenced by mechanical stiffness of the TME and understand CAF-cancer cell crosstalk. Analyses presented here were conducted using Illumina RNA-seq along with a bioinformatic algorithm described later to ensure sequence quality.

## Materials and Methods

### Substrate preparation

Substrates with different elastic moduli (1, 10 and 40 kPa) were prepared with Polyacrylamide (PA) gel layered on top of glass-bottom petri dishes (Cellvis). First, the glass surfaces were silanized with (3-Aminopropyl)trimethoxysilane (APTES) solution (Sigma-Aldrich) for 5 mins. Next, the silanized surfaces were functionalized with 0.5% glutaraldehyde solution (Polysciences) for 30 mins. PA gel precursor solutions were prepared by mixing Acrylamide (40% sol., Sigma-Aldrich), N,N′-Methylenebis- acrylamide (2% sol., Sigma-Aldrich) and Phosphate Buffered Saline (PBS, Corning) at specific ratios according to the protocol^9,10^. Addition of 1% (v/v) of ammonium persulfate (APS) solution (10% w/v APS, Bio-Rad) and 0.1% (v/v) of Tetramethylethylenediamine (TEMED) solution (Bio-Rad) starts the polymerization of the gel. For a 30 mm diameter surface, 95 ul PA gel solution is added to bottom glasses, and then sandwiched with clean coverslips. After polymerization, the top coverslips were removed and 0.2 mg/ml sufosuccinimidyl-6- (4′-azido- 2′-nitrophenylamino)- hexanoate (Sulfo-SANPAH, ThermoFisher Scientific) solution in HEPES buffer (50 mM HEPES at pH 8.5, ThermoFisher Scientific) was applied. Next, activation with 365 nm UV light (8 Watt, UVP UVL-28, Analytik Jena, US) for 20 mins binds the Sulfo-SANPAH cross-linkers to the PA gel. For ECM functionalization, 25 µg/ml Fibronectin (Human, Corning) solution in HEPES buffer was prepared and the substrates were soaked in the ECM solution overnight. After ECM coating, 3 times rinsing with PBS prepared the substrates for cell plating.

### Cell culture

Human primary colorectal tumor CAFs, CAF05 (Neuromics), were maintained in VitroPlus III Low Serum, Complete medium (Cat. # PC00B1, Neuromics) supplemented with 1% penicillin-streptomycin (Lonza). Cells were grown at 37 °C in a humidified incubator with 5% CO_2_. We used the CAFs with low passage numbers (passage 3–9), and the CAFs are alpha-SMA positive. This indicates that this population does not have any epithelial cancer cells. The culture media was not supplemented with exogenous TGF-beta or Matrigel. However, the substrates were coated with fibronectin for cell attachment. Other relevant metadata is presented in Table 1.

**Table 1:** Clinical information on CAF05.

Cell	Tissue Type	Tumor Stage	Grade	T/N/M	Donor ID	Sex	Age	Race	Treatment
CAF05	Colo- rectal tumor	N/A	N/A	N/A	BTC1000-E1110035885100614HS	Male	55	Caucasian	N/A

### RNA extraction and sequencing

After 48 hours of culture on substrates of specified stiffness, RNA was extracted using Trizol reagent (Invitrogen) as per manufacturer instructions. Samples for RNA-seq were further cleaned up by RNeasy Mini Kit (QIAGEN). The RNA-seq libraries were prepared with Illumina TruSeq Stranded mRNAseq Sample Prep kit (Illumina). Paired-end, polyA + RNA-sequencing was performed on Illumina platform at the Roy J. Carver Biotechnology Center at UIUC.

### Quality validation and RNA-seq analysis

The quality of the RNA-seq libraries were evaluated using FastQC (http://www.bioinformatics.babraham. ac.uk/projects/fastqc/). The reads were aligned to the human genome assembly GRCh38.p13 using HISAT2^11^. The gene counts were first quantified using HTseq-Count^12^, then the gene expression was analyzed using edgeR^13^. Normalization of library size was performed by calcNormFactors in edgeR with the default TMM method. Heatmaps were plotted using the coolmap function from the limma package^14^ with row centering and scaling. Hierarchical clustering of genes was performed with average-linkage method. Differentially expressed genes were defined by |log2(fold change)| >1 and FDR <0.05. Gene ontology analyses, GSEA (gene set enrichment analysis), and disease ontology analysis were performed using clusterProfiler of Bioconductor^15,16^.

## Data Records

Data from the RNA-seq were deposited to the NCBI Gene Expression Omnibus (GEO) under accession number GSE229742^17^.

## Technical validation

### Library quality

RNA quality was assessed using Agilent Fragment Analyzer in the Roy J. Carver Biotechnology Center, and all samples were determined to be suitable for poly (A) selection and sequencing. 49 to 87 million poly (A)-selected paired-end sequence reads were obtained per sample from Illumina NovaSeq 6000 in the Roy J. Carver Biotechnology Center (Fig. 2A). The quality of the RNA-seq libraries were evaluated using FastQC (http://www.bioinformatics.babraham. ac.uk/projects/fastqc/).

### Quality validation

The mean Phred quality score across the reads were evaluated and plotted by FastQC, which shows that all samples have a mean per base Phred score >34 (Fig. 2C). The Phred quality score per sequence also shows that the sequencing reads have an overall high quality, Phred score >28 (Fig. 2D). Principal Component Analysis (PCA) shown in Fig. 2B confirms the similarity between most biological replicates PC1 explains 42.62% of the variance and PC2 explains 23.65% of the variance. The GC contents of the samples are also within 40–50% (Fig. 2E).

### Immunostaining

Cells were fixed, permeabilized and blocked with 4% Paraformaldehyde (PFA) in PBS (30 mins), 0.2% Triton X-100 in PBS, and 2.5% bovine serum albumin (BSA) with 2% normal goat serum (NGS) in PBS. Following overnight incubation in αSMA antibody (1:500) (Sigma, cat. no. A5228), the samples were incubated with Alexa Fluor 568 conjugated secondary antibody (1:1000) (Abcam Inc., cat. no. ab175695) at 4 °C for 12 hrs. Next, the samples were incubated in 4′,6-diamidino-2-phenylindole (DAPI) (1:1000) (Invitrogen, cat. no. D1306) for 10 minutes and imaged with LSM710 (Zeiss) confocal microscope using an EC Plan-Neofluar 20X/0.5 NA objective lens (Zeiss).

## Usage Notes

The RNA-seq data shared in this article can be processed using a collection of open access tools. For instance, the raw fastq data can be aligned to human reference genome assembly (e.g. GRCh38.p13) using aligners such as STAR^11^ and HISAT2^18^. For this study, we used the HISAT2 aligner. Other compatible aligners can also be used for this purpose and alignment can be accessed by various genome browsers such as ZENBU^19^, Integrative Genome Viewer (IGV)^20^ or UCSC Genome Browser^21^. Differential gene expression analysis can be performed using publicly available packages such as edgeR^13^, DESeq 2^22^ and CuffDiff2^23^. We have used edgeR with TMM normalization to analyze the differentially expressed genes from various mechanical stiffness of the substrates and performed hierarchical clustering of genes with average-linkage method. Gene ontology (GO) analyses, GSEA (gene set enrichment analysis), and disease ontology analysis can be performed using clusterProfiler of Bioconductor^15,16^. It should be noted that there are methods available for alignment-free differential gene expression analysis. For such applications, transcript quantification can be performed using Sailfish^24^ or Kallisto^25^. Gene-level abundance estimates, and statistical inference can be made using packages such as tximport^26^ and then differential expression can be determined with DESeq 2 or edgeR.

**Fig. 3:**
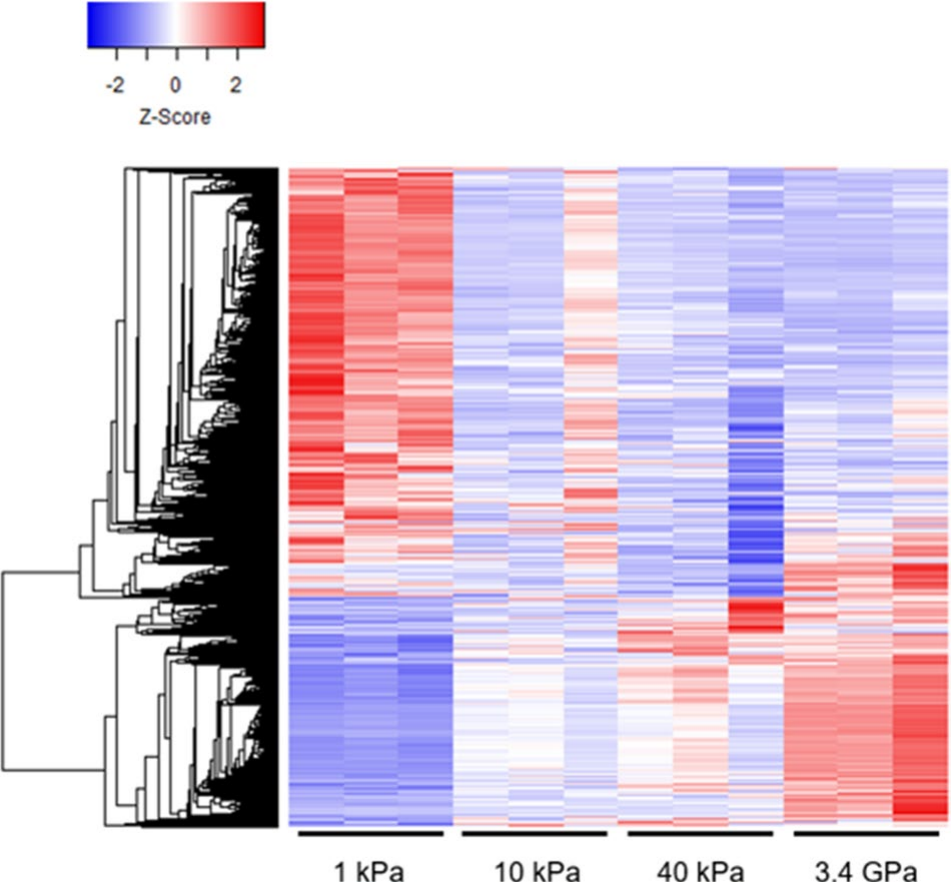
CAFs undergo significant gene expression changes when grown on substrates of increasing stiffness. Hierarchical clustered heatmaps showing the relative expression levels of differentially expressed genes based on RNA-seq. Rows represent genes which are hierarchically clustered using the average-linkage clustering method. Columns represent the samples, showing three biological replicates of CAFs grown at 1 kPa, 10 ka, 40 kPa, and 3.4 GPa of stiffness.

Data shared here provides insights into the biological processes that take place in CAFs while they adapt to increasing rigidity in the tumor microenvironment. Although our cells are taken from colorectal cancer, these results should also be relevant to other solid form of tumors such as breast, prostate and lung cancers. Also, the culture conditions do not allow cross-talk between CAFs and cancer cells. Hence, these results strictly represent the effects of mechanical stimulation of the CAFs. Hierarchical clustered heatmaps show that stiffness alone can result in differential expression of many genes (Fig. 3). Gene ontology (GO) analyses and gene set enrichment analyses (GSEA) show that substrate stiffness has profound influence on many signaling pathways such as chromatin assembly, nuclear organization, cell membrane function and cytoskeleton organization in CAFs. Further analysis of the data is required to identify novel pathways and biomarkers in cancer development and metastatic progression. In addition, this data can be compared with RNAseq data from primary cancer cells to identify pathways that may be regulated by stiffness-dependent cross-talk between CAFs and cancer cells. This data may also provide guidance for choosing biomechanics based therapeutic targets. As a result, we are publicly sharing this data for researchers in relevant fields to encourage further reuse of the transcriptomics.

## Data Availability

The following software and versions were used for quality control and data analysis: 1. FastQC, version 0.11.8 and MultiQC, version 1.7 were used for quality analysis of raw FASTQ sequencing data: http://www.bioinformatics.babraham. ac.uk/projects/fastqc/ 2. HISAT2 was used for mapping of sequence reads to the human GRCh38.13 genome assembly: http://www.ccb.jhu.edu/software/hisat/index.shtml 3. HTSeq, version 0.9.1 was used for calculating the gene counts: http://bioinf.wehi.edu.au/featureCounts/ 4. edgeR, version 3.34.1 was used for normalization and visualization of differential gene expression analysis output: https://bioconductor.org/packages/release/bioc/html/edgeR.html Software and codes are open source and readily available.
